# Supplementary material for: Role of Human DNA Ligases in Mediating Pharmacological Activities of Flavonoids
Source: Int J Mol Sci. 2025 Feb 10;26(4):1456. doi: 10.3390/ijms26041456 (PMC11855144; doi:10.3390/ijms26041456)
Supplement: Supplementary file 1 [file ijms-26-01456-s001.zip › ijms-3412969-supplementary.pdf]

Supplementary data for

Role of Human DNA Ligases in Mediating Pharmacological Activities of Flavonoids

Daekyu Sun [1,2,*] and Vijay Gokhale [2,3]

[1] Department of Pharmacology and Toxicology, College of Pharmacy, University of Arizona, Tucson, AZ 85721, USA

[2] Bio5 Institute, University of Arizona, Tucson, AZ 85721, USA; gokhale@reglagene.com

[3] Reglagene, Inc., Tucson, AZ 85719, USA

* Correspondence: sun@pharmacy.arizona.edu

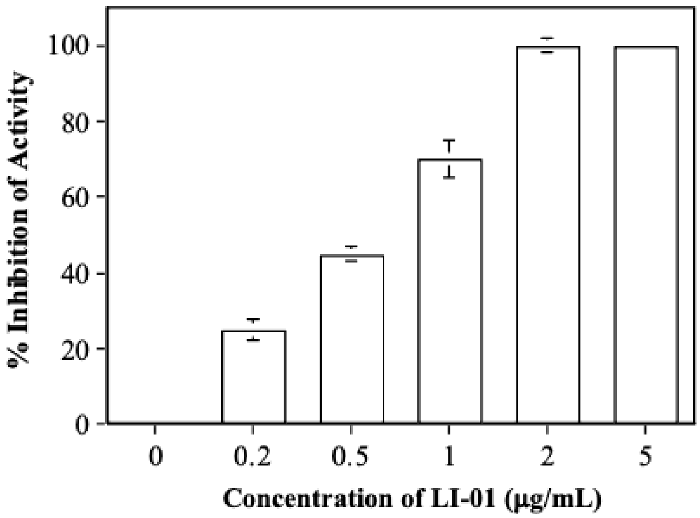

Figure S1. Graphical representation of Figure 1. Data shown are expressed as the mean of at least two independent experiments; error bars indicate the standard error of the mean.

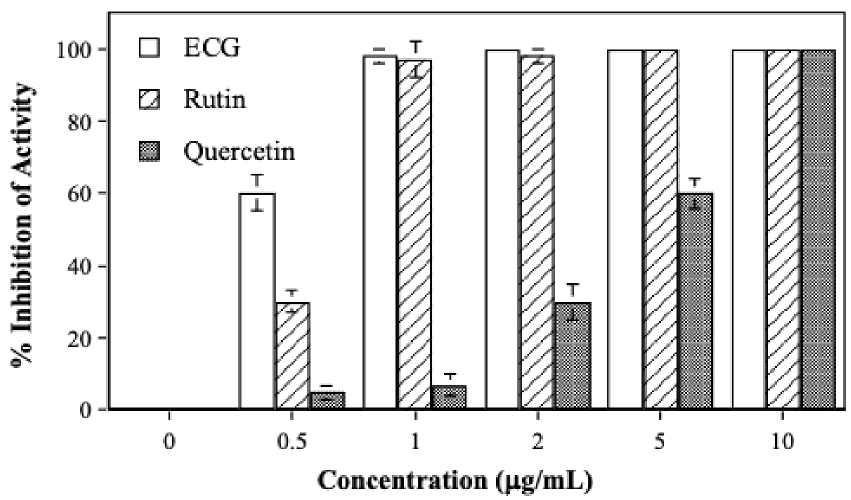

Figure S2. Graphical representation of Figure 2. Data shown are expressed as the mean of at least two independent experiments; error bars indicate the standard error of the mean.

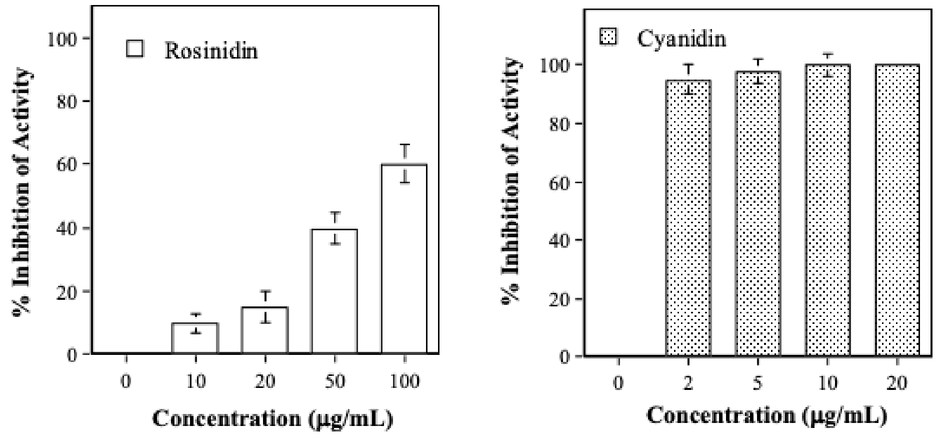

Figure S3. Graphical representation of Figure 3. Data shown are expressed as the mean of at least two independent experiments; error bars indicate the standard error of the mean.

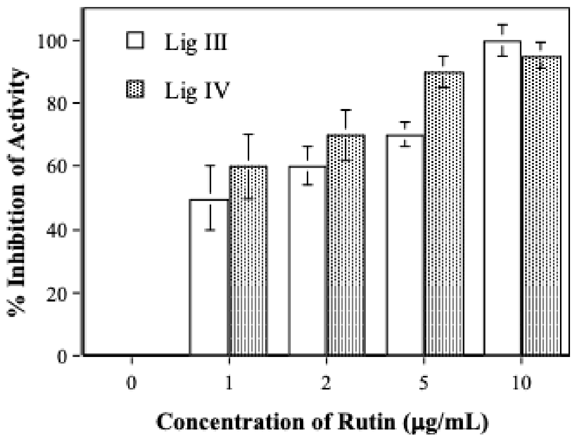

Figure S4. Graphical representation of Figure 5. Data shown are expressed as the mean of at least two independent experiments; error bars indicate the standard error of the mean.
